# Predicting clinical outcome with phenotypic clusters using quantitative CT fibrosis and emphysema features in patients with idiopathic pulmonary fibrosis

**DOI:** 10.1371/journal.pone.0215303

**Published:** 2019-04-18

**Authors:** So Hyeon Bak, Hye Yun Park, Jin Hyun Nam, Ho Yun Lee, Jeong Hyun Lee, Insuk Sohn, Man Pyo Chung

**Affiliations:** 1 Department of Radiology and Center for Imaging Science, Samsung Medical Center, Sungkyunkwan University School of Medicine, Seoul, Korea; 2 Department of Health Sciences and Technology, SAIHST, Sungkyunkwan University, Seoul, Korea; 3 Department of Radiology, School of Medicine, Kangwon National University, Chuncheon, Republic of Korea; 4 Division of Pulmonary and Critical Care Medicine, Department of Medicine, Sungkyunkwan University School of Medicine, Seoul, Republic of Korea; 5 Department of Public Health Sciences, Medical University of South Carolina, Charleston, SC, United States of America; 6 Statistics and Data Center, Research Institute for Future Medicine, Samsung Medical Center, Seoul, Republic of Korea; National and Kapodistrian University of Athens, SWITZERLAND

## Abstract

**Background:**

The clinical course of IPF varies. This study sought to identify phenotyping with quantitative computed tomography (CT) fibrosis and emphysema features using a cluster analysis and to assess prognostic impact among identified clusters in patient with idiopathic pulmonary fibrosis (IPF). Furthermore, we evaluated the impact of fibrosis and emphysema on lung function with development of a descriptive formula.

**Methods:**

This retrospective study included 205 patients with IPF. A texture-based automated system was used to quantify areas of normal, emphysema, ground-glass opacity, reticulation, consolidation, and honeycombing. Emphysema index was obtained by calculating the percentage of low attenuation area lower than -950HU. We used quantitative CT features and clinical features for clusters and assessed the association with prognosis. A formula was derived using fibrotic score and emphysema index on quantitative CT.

**Results:**

Three clusters were identified in IPF patients using a quantitative CT score and clinical values. Prognosis was better in cluster1, with a low extent of fibrosis and emphysema with high forced vital capacity (FVC) than cluster2 and cluster3 with higher fibrotic score and emphysema (*p* = 0.046, and *p* = 0.026). In the developed formula [1.5670—fibrotic score(%)*0.04737—emphysema index*0.00304], a score greater ≥ 0 indicates coexisting of pulmonary fibrosis and emphysema at a significant extent despite of normal spirometric result.

**Conclusions:**

Cluster analysis identified distinct phenotypes, which predicted prognosis of clinical outcome. Formula using quantitative CT values is useful to assess extent of pulmonary fibrosis and emphysema with normal lung function in patients with IPF.

## Introduction

Idiopathic pulmonary fibrosis (IPF) is the most common form of chronic, progressive, interstitial pneumonia with restrictive ventilator dysfunction and reduced gas exchange [1, 2]. The prognosis of IPF is poor overall, with mean survival ranging from 2.5 to 5 years after definite diagnosis [3]. However, the clinical course of IPF varies substantially [4]. Predicting the clinical course or outcome for an individual patient is important but difficult. Clinical variables correlated with survival include age, sex, smoking status, dyspnea, pulmonary functions, digital clubbing, body mass index, and pulmonary hypertension [5]. Computed tomography (CT) plays a central role in the systemic assessment of patients with suspected IPF [6]. The extent of lung fibrosis on CT correlates with disease severity and mortality in interstitial lung disease [4, 7, 8]. Visual scoring of IPF by radiologists is limited by the availability of specialist radiologists and high interobserver variability and is somewhat subjective. There is moderate interobserver agreement among radiologists in identifying honeycombing, which is a diagnostic criterion of IPF [9]. Compared with visual assessment, quantitative analysis of IPF offers an objective, detailed, and reproducible measurement of the extent of IPF [1, 10].

As for spirometry, forced vital capacity (FVC) has been identified as an indicator of disease progression in IPF and is widely used for the routine monitoring of IPF and as a primary endpoint in drug trials [11–13]. Meanwhile, approximately one-third of patients with IPF also have emphysema, and have a normal lung volume as the lung volume increases by 5–10% [11, 13, 14]. Furthermore, higher mortality and less extensive fibrosis have been reported in the patients with IPF combined with emphysema at diagnosis than those with IPF alone [13, 14]. Therefore, when emphysema coexists with pulmonary fibrosis, spirometric values may be misinterpreted as normal.

Cluster analysis has been used to identify homogeneous phenotypic clusters in patients with chronic obstructive pulmonary disease, interstitial lung disease (ILD), and asthma [15–17]. The purpose of our study was two-fold. Firstly, we sought to identify radiologic-based phenotyping with a quantitative CT fibrotic score and emphysema features using a cluster analysis in IPF patients, ultimately reflecting prognostic differences among the identified clusters. Secondly, we evaluated the impact of fibrosis and emphysema on lung function in IPF patients with development of a descriptive formula.

## Materials and methods

### Study population

**Fig 1:**
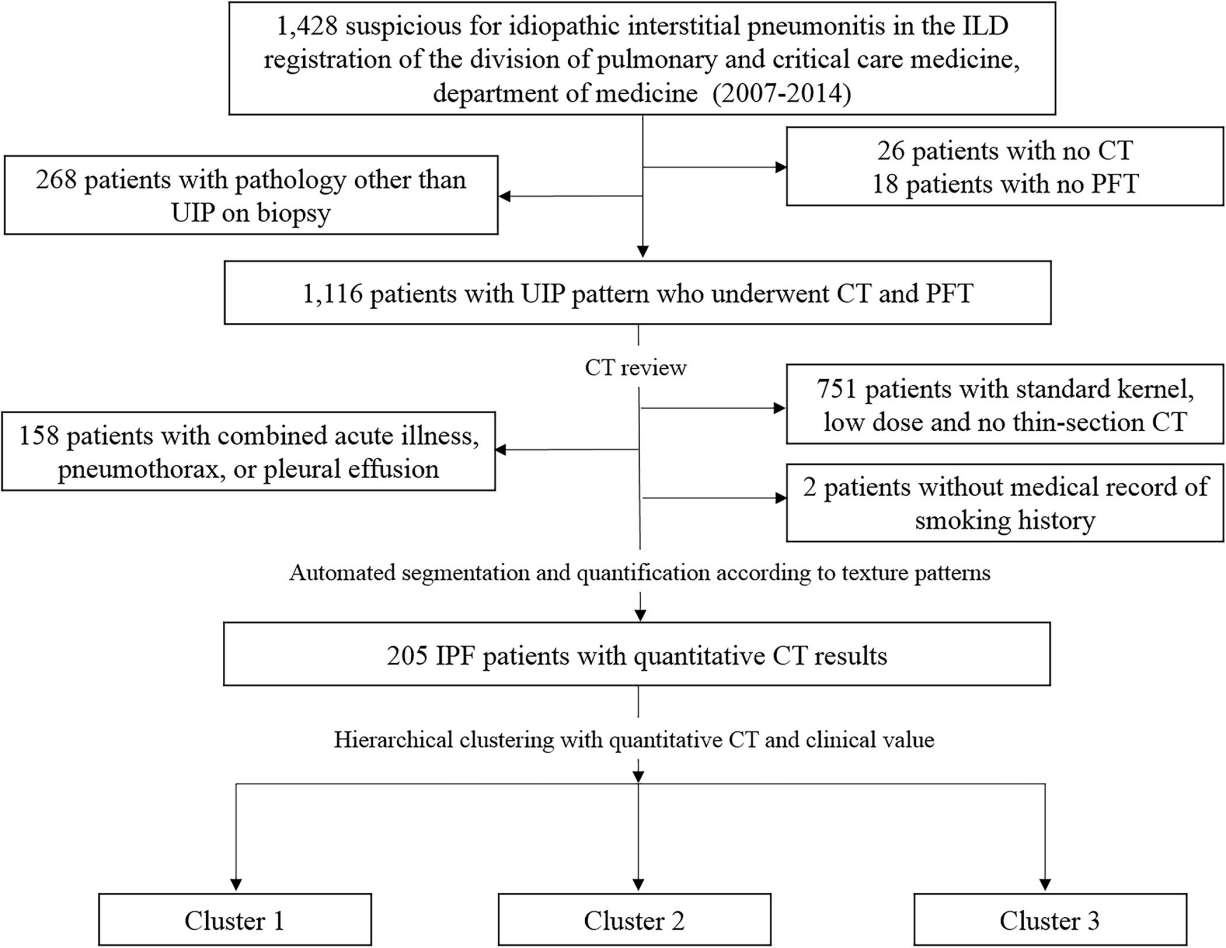
Flow chart of study design. UIP = usual interstitial pneumonitis; IPF = idiopathic pulmonary fibrosis; CT = computed tomography.

The Institutional Review Board of our institute approved this retrospective study (approval #2013-09-119), and the requirement for informed consent was waived. From 2007 to 2014, 1117 patients were diagnosed with IPF and underwent CT and pulmonary function test (PFT). The usual interstitial pneumonia (UIP) diagnosis was based on the American Thoracic Society/European Respiratory Society (ATS/ERS) statement with the presence of a UIP pattern on CT in patients not subjected to surgical lung biopsy or specific combinations of CT and surgical lung biopsy patterns in those who received a surgical lung biopsy [2, 18]. Nine hundred nine patients were excluded from our study, including 158 patients with combined acute illness, pneumothorax, or pleural effusion and 751 patients who underwent CT with a standard kernel, low dose, or thick section protocol. We further excluded 2 patients without medical record of smoking history. Final sample included 205 IPF patients with volumetric thin-section CT and PFT (Fig 1).

### Texture-based automated quantification

**Fig 2:**
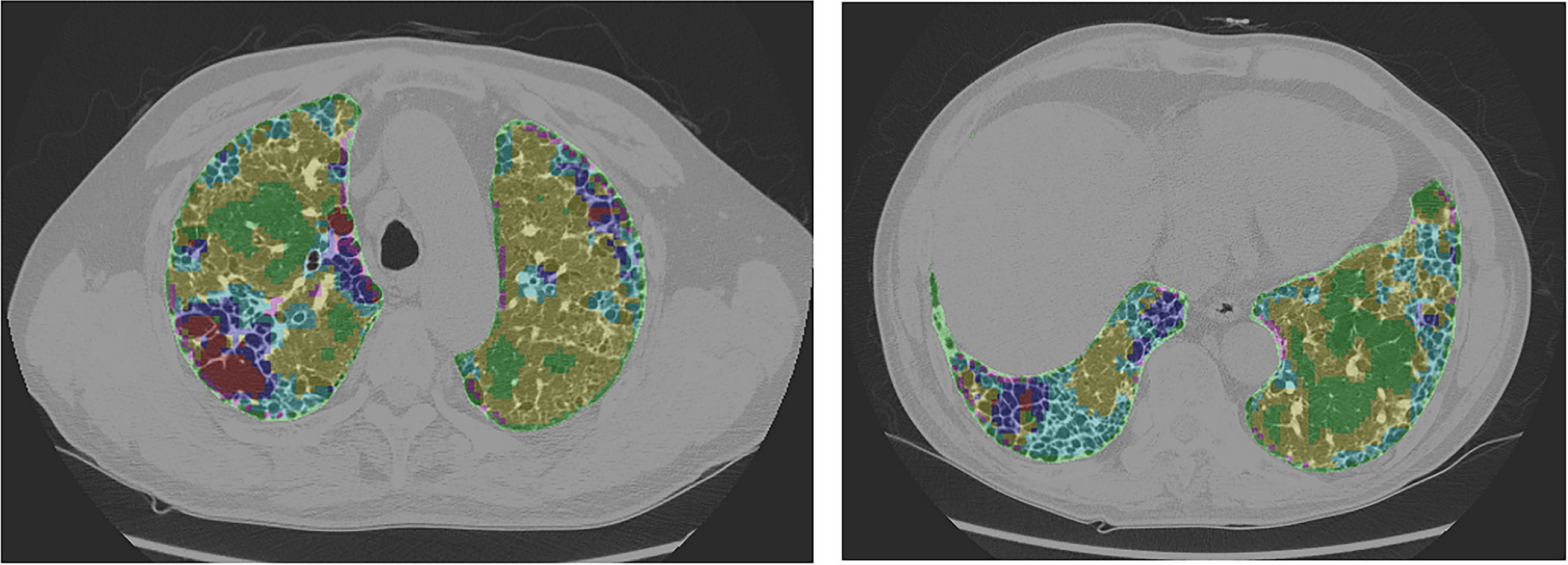
Texture-based automated quantification was performed on volumetric thin-section CT in patients with IPF and shows the extent of regional patterns with different colors. Green = normal, yellow = ground-glass opacity, cyan = reticulation, red = emphysema, pink = consolidation, blue honeycombing. IPF = idiopathic pulmonary fibrosis; CT = computed tomography.

In-house software was developed for texture-based automated quantification using Visual C++ (Visual Studio 2013, Microsoft) and the Insight Segmentation and Registration Toolkit (ITK, version 4.7) (National Library of Medicine). Whole-lung segmentation was automatically extracted. Based on the texture-based automated system, six regional patterns of normal, reticulation, ground-glass opacity (GGO), consolidation, honeycombing, and emphysema were automatically quantified (Fig 2). Fibrotic score (FS) was defined as the sum of the extent of fibrosis-associated GGO, reticulation, and honeycombing. The methods of texture-based automated quantification have been previously described in detail [10]. Emphysema index was obtained by calculating the percentage of the low attenuation area lower than -950HU.

### Statistical analysis

Parametric data are expressed as mean ± standard deviation (SD), whereas non-parametric data are expressed as numbers and percentages. Quantitative CT and clinical characteristics including patterns for normal, reticulation, consolidation, GGO, and honeycombing, age, sex, smoking, and PFT were divided into three clusters by Consensus Clustering with the agglomerative hierarchical clustering algorithm using Spearman correlation distance. Differences in clinical and quantitative CT values among the three clusters were tested using Fisher’s exact test, Chi-square test, ANOVA test, and Kruskal-Wallis test. Survival curves were estimated using Kaplan-Meier methods, and survival analysis was performed using Cox regression with Bonferroni’s correction. Two-sided P-values less than 0.05 were considered significant.

For the formula to evaluate the impact of fibrosis and emphysema on pulmonary function in patients with IPF, we considered several methods, including linear discriminant analysis, logistic regression, cut-off criterion, and support vector machines (SVMs). Ultimately, SVMs were confirmed based on cross-validation (CV) accuracy. SVMs separate the data into different classes using a hyper-plane **w**·**x**+b = 0 corresponding to the decision function f(**x**) = sign(**w**·**x**+b), which minimizes ${\frac{1}{2}|\left|w\right||}^{2}+C\sum_{i}^{n}\xi_{i}$ subject to y_i_(***w***·***x***_*i*_+*b*)≥1−*ξ*_*i*_,*ξ*_*i*_≥0, where ξ_i_ is a slack variable, and *C* is a tuning parameter. Here, a category of normal or restrictive was used as response variable y, and FS (%) and emphysema index were used as **x**. To make a decision function using SVMs, kernel function and tuning parameter *C* were needed a priori. Based on 100 repetitions of 5-fold CV, we selected a linear kernel and *C* = 8, which maximized CV accuracy. For the linear kernel function, polynomial, sigmoid, and Gaussian RBF were considered, with the range of *C* from 0 to 10. To estimate the formula, the R package “kernlab” was used. Data analysis was performed using R 3.4.3 (Vienna, Austria; http://www.R-project.org/) and SAS version 9.4 (SAS Institute Inc., Cary, NC).

## Results

### Demographic characteristics

Of the 205 included patients, 78.0% were male, the mean age was 65.8 years (range; 45 to 96 years), 31.2% performed surgical lung biopsy, the mean FVC was 79.0%, and mean diffusing capacity of the lung carbon monoxide (DLco) was 62.9%. When assessing fibrosis and emphysema on CT with texture-based automated quantification, the mean score was 3,794,216.5 mm^3^ for whole lung volume, 44,974.8 mm^3^ for honeycombing, 278,098.0 for reticulation, 776,471.6 for GGO, and 44,003.8 for consolidation. The mean emphysema index was 5.6. Of the 205 patients, 17 (8.3%) had an obstructive and combined pattern, 93 (45.4%) had a normal pattern, and 95 (46.3%) had a restrictive pattern in PFT. Characteristics of the 205 patients are summarized in Table 1.

**Table 1:** Characteristics of 205 patients with IPF.

Characteristics	Value
Age	65.8 ± 7.6
Sex (male)*	160 (78.0)
Pulmonary Function Tests	
FVC(%)	79.0 ± 17.6
FEV_1_ (%)	89.3 ± 19.3
FEV_1_/FVC ratio	79.9 ± 7.1
DL_CO_ (%)	62.9 ± 18.9
TLC (%)	77.6 ± 14.8
CPI	38.5 ±14.5
Pattern (mm^3^)	
Whole lung volume	3,797,216.5 ± 986,814.5
Normal	2,588,276.5 ± 1,306,646.9 (68.2%)
Honeycombing	44,974.8 ± 86,107.1 (1.2%)
GGO	776,471.6 ± 497,800.3 (20.5%)
Consolidation	44,003.8 ± 23,634.1 (1.2%)
Reticulation	278,098.0 ± 304,913.2 (7.3%)
Emphysema index	5.6 ± 4.6

CPI = composite physiologic index; DL_CO_ = diffusing capacity of the lung for carbon monoxide; FEV_1_ = forced expiratory volume in 1 second; FVC = forced vital capacity; IPF = idiopathic pulmonary fibrosis; GGO = ground-glass opacity

Data are shown as mean ± standard deviation, and data in parentheses are percentages.

*Data are numbers of male and data in parentheses are percentages.

### Clustering analysis

**Fig 3:**
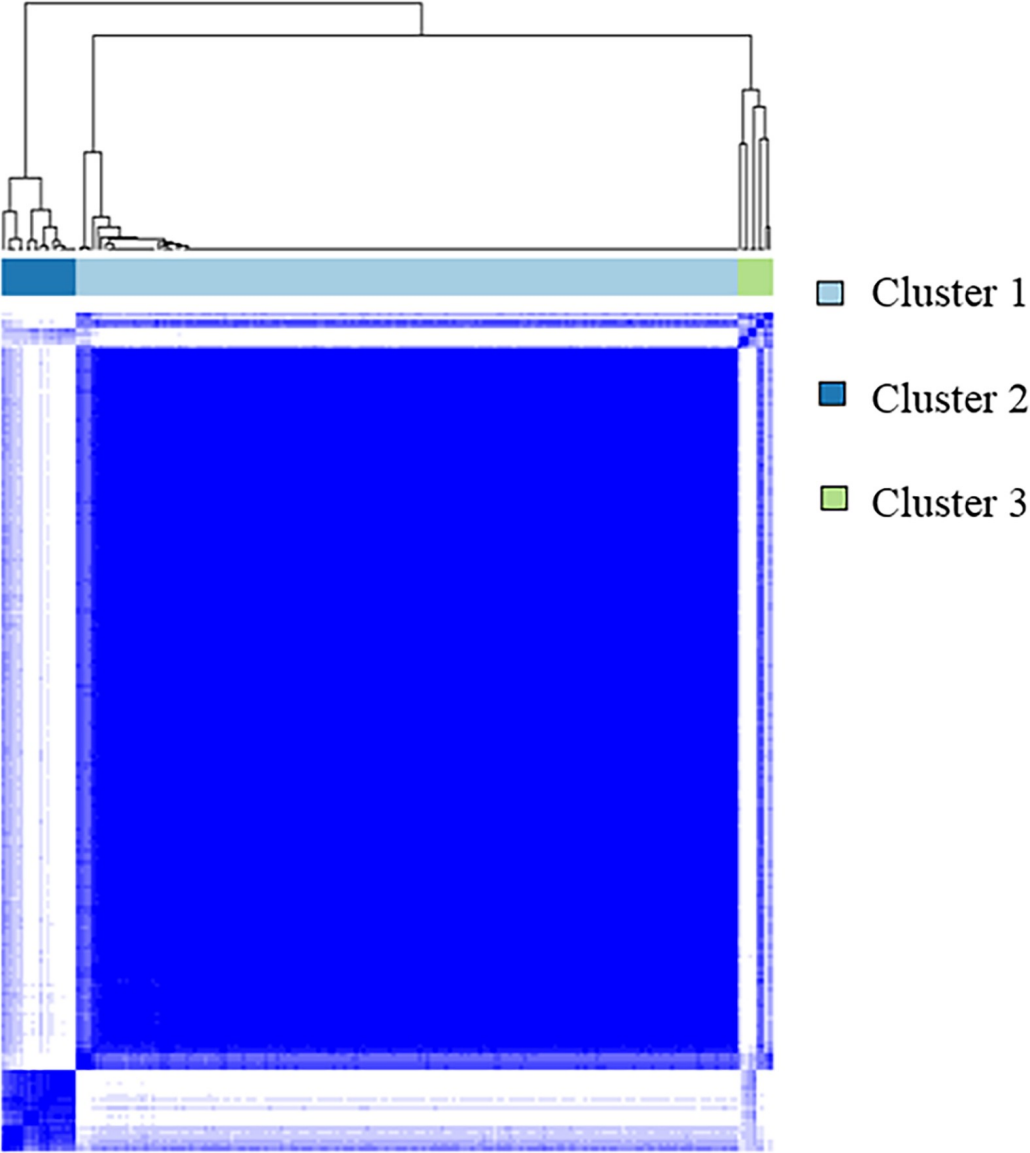
Consensus Cumulative Distribution Function (CDF) Pot using quantitative CT and clinical values.

**Fig 4:**
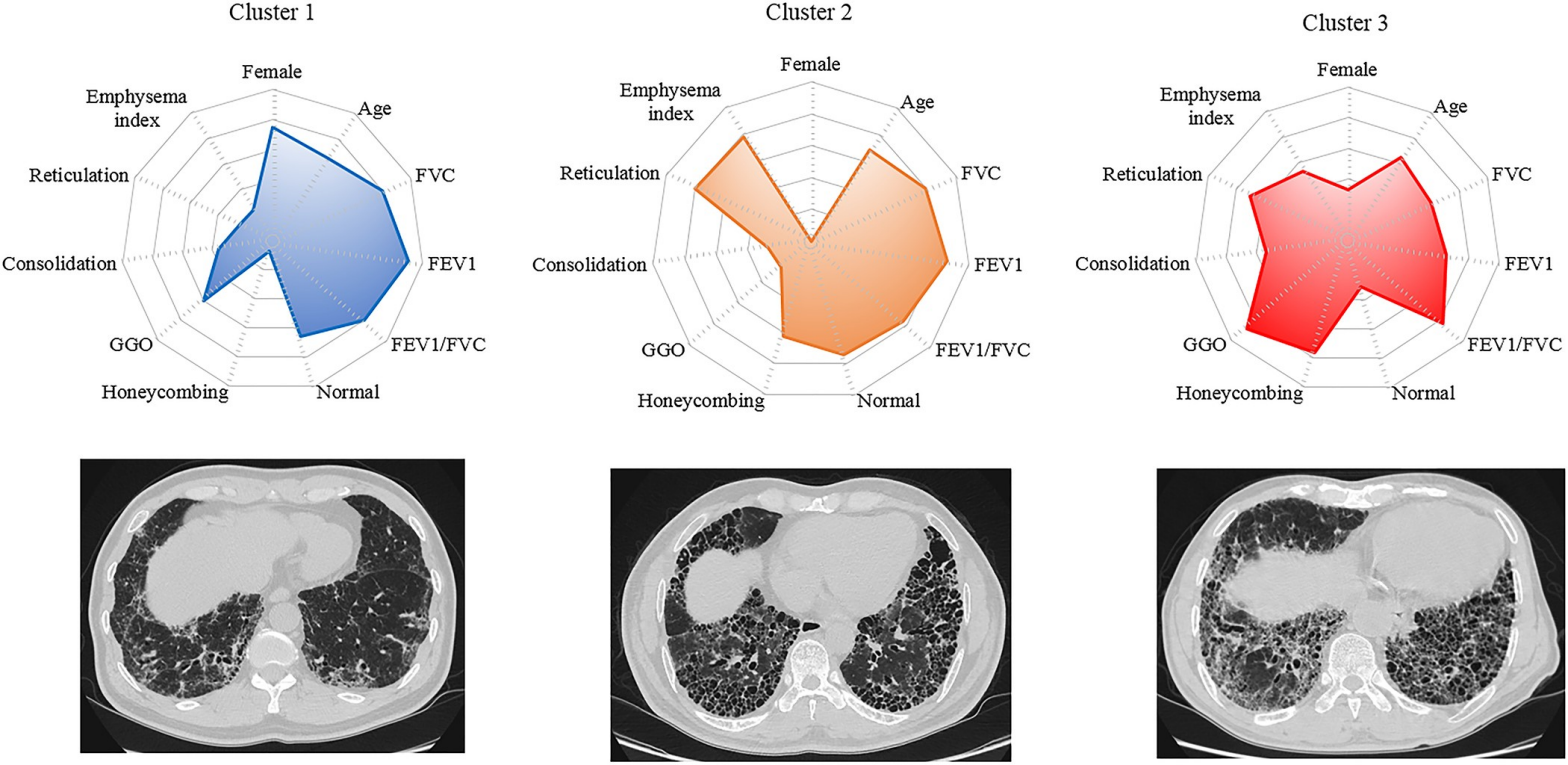
Radar plot according to phenotypic clusters (top) and CT images corresponding to each clusters (bottom).

We performed Consensus hierarchical clustering with quantitative CT and clinical features for 205 patients. Consensus clustering analysis was used to divide the patients into three clusters (Fig 3). Subjects grouped into cluster 1 tended to be female and had higher FVC on PFT. The prevalence of reticulation, honeycombing, and emphysema was low, while the prevalence of GGOs was high. Subjects in cluster 2 were predominantly male and had higher FVC on PFT, along with a high prevalence of honeycombing, reticulation, and emphysema, but were the lowest for GGOs on CT. Subjects in cluster 3 had the lowest FVC on PFT; the greatest prevalence of honeycombing, consolidation, and GGOs; and the lowest normal volume on CT. DLco was 64.8% in cluster 1, 52.8% in cluster 2, and 42.3% in cluster 3. Cluster 1 had significantly lower DLco than cluster 2 (*p* = 0.023) and cluster 3 (*p* = 0.005). However, there was no significant difference in DLco between cluster 2 and 3 (*p* = 0.403). Sixty-three of the 176 patients in cluster 1, 0 in 90 patients in cluster 2, and 1 in 9 in cluster 3 were diagnosed as IPF by surgical lung biopsy. A summary of the comparisons among the three clusters is shown in Table 2 and Fig 4.

**Table 2:** Characteristics of the three clusters according to Consensus clustering using quantitative CT and clinical features.

Characteristics	Cluster 1 (n = 176)	Cluster 2 (n = 20)	Cluster 3 (n = 9)	P-value	P-value†	P-value‡	P-value§
Sex (male)*	132 (75%)	20 (100%)	8 (88.9%)	0.015	0.026	1	0.931
Age	65.5 ± 7.6	68.6 ± 6.7	64.9 ± 8.2	0.273			
Smoking status				0.247			
Current smoker	15 (8.5)	4 (20)	1 (11.1)				
Ex-smoker	93 (52.8)	12 (60)	4 (44.4)				
Non-smoker	68 (38.7)	4 (20)	4 (44.4)				
Pulmonary Function Tests							
FVC	79.7 ± 17.5	79.2 ± 14.4	63.8 ± 20.9	0.029	1	0.027	0.086
FEV_1_	90.5 ± 19.6	86.6 ± 12.8	72.9 ± 18.9	0.023	1	0.029	0.088
FEV_1_/FVC	80.1 ± 6.5	76.7 ± 9.3	82.7 ± 11.2	0.157			
Pattern (mm^3^)							
Normal	2,613,015.9 ± 1,325,027.6	2,972,241.2 ± 983,977.0	1,251,227 ± 661,978.1	0.003	0.724	0.008	< .0001
Honeycombing	15,657.6 ± 25,565.1	208,361.6 ± 111,838.9	255,208.6 ± 98,461.7	< .0001	< .0001	< .000	0.688
GGO	807,501.4 ± 495,473.7	326,882.0 ± 235,448.5	1,168,755.0 ± 343,862.6	< .0001	< .0001	0.042	0.688
Consolidation	44,132.5 ± 23,557.5	35,070.8 ± 18,399.7	61,336.6 ± 27,604.0	0.024	0.266	0.129	0.031
Reticulation	245,720.0 ± 259,780.7	483,797.7 ± 538,742.5	454,157.5 ± 203,685.5	0.000	0.029	0.005	0.744
Emphysema index	4.6 ± 3.4	13.4 ± 5.9	9.0 ± 4.1	< .0001	< .0001	0.004	0.121

FEV_1_ = forced expiratory volume in 1 second; FVC = forced vital capacity; GGO = ground-glass opacity

Data are shown as mean ± standard deviation

*Data are numbers of male, and data in parentheses are percentages.

†P-value indicates difference between cluster 1 and cluster 2.

‡ P-value indicates difference between cluster 1 and cluster 3.

§ P-value indicates difference between cluster 2 and cluster 3.

### Survival

**Fig 5:**
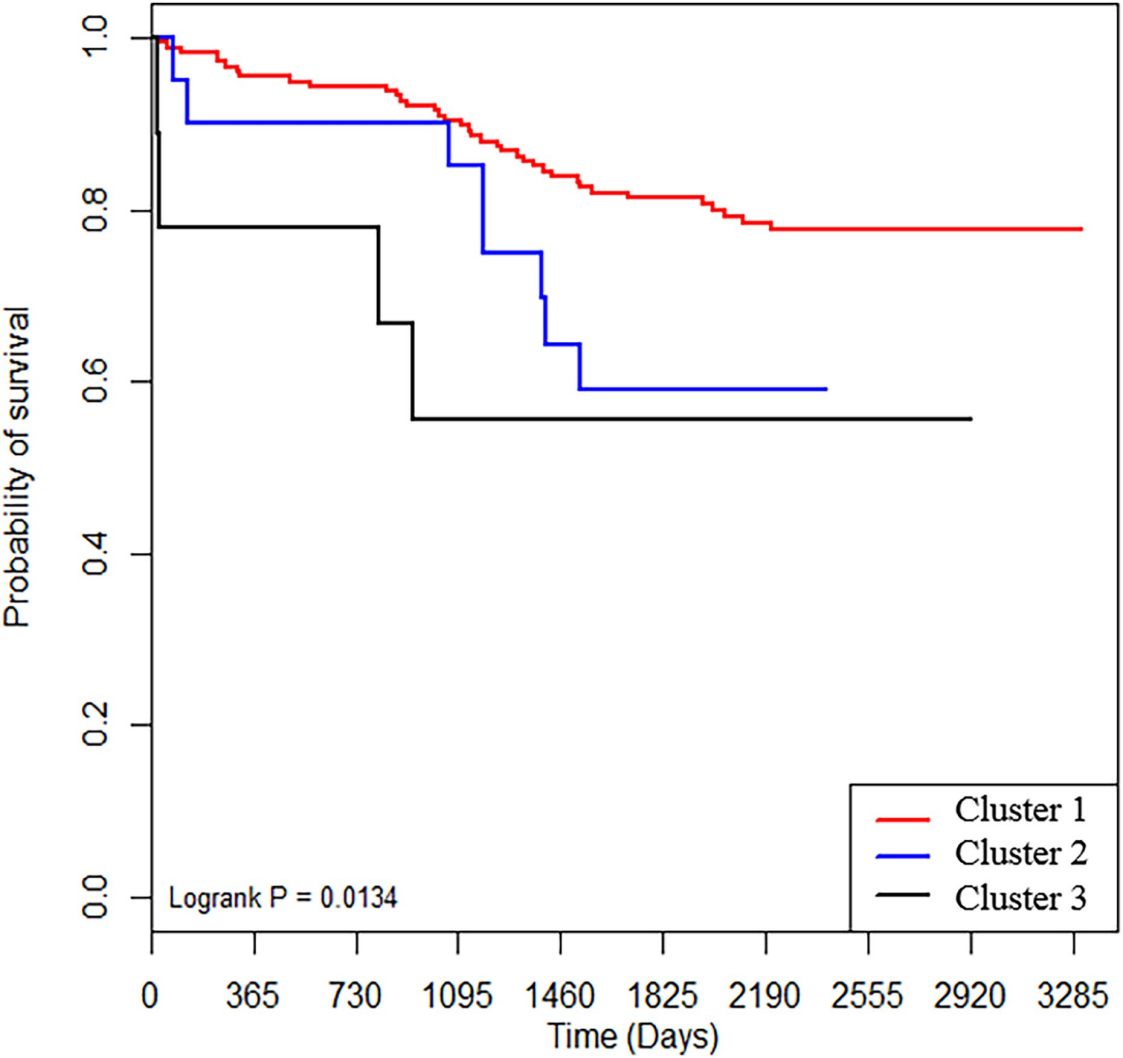
Survival in patients with IPF. Patients were substratified according to Consensus clustering. IPF = idiopathic pulmonary fibrosis.

Survival was evaluated according to IPF classification. There were significant differences in survival among the three clusters by Consensus clustering (*p* = 0.019). Cluster 1, with a low fibrotic score and high FVC, showed good prognosis when compared with cluster 2 (*p* = 0.046) and cluster 3 (*p* = 0.026) with higher fibrotic scores. Cluster 3 had a worse prognosis than cluster 2, but the difference was not significant (*p* = 0.520) (Fig 5).

### Formula based on quantitative CT for assessing impact of fibrosis and emphysema

Of the 205 patients, except for 17 with an obstructive and combined pattern on PFT, 188 patients were divided into two subgroups according to the results of the PFT: restrictive (95 patients) and normal pattern (93 patients). A formula reflecting spirometric results was derived using FS and emphysema index on quantitative CT in patients with IPF. The FS was different between the restrictive and normal subgroups based on PFT, and the restrictive subgroup had higher FS than those in the normal subgroup (restrictive 43.4 ± 20.5, normal 24.3 ± 17.8; *p* = 0.000). However, emphysema index was not significantly different between the two groups (restrictive 5.7 ± 3.9, normal 4.9 ± 4.7; *p* = 0.210). The formula to assess the extent of FS and emphysema in patients with IPF is as follows: 1.5670—FS(%)*0.04737—emphysema index*0.00304. A score greater than 0 indicates coexistence of pulmonary fibrosis and emphysema as a significant extent despite of normal spirometric result. The accuracy of the formula which is the probability that the PFT normal or restrictive patients are exactly classified by formular was 0.67, and the true positive rate (TPR) which is the probability that the PFT normal patients are classified as normal was 0.71.

## Discussion

In the present study, quantitative CT features dealing both with fibrotic score and emphysema index were used for clustering of radiologic phenotyping in patients with IPF, yielding three clusters. The radiologic phenotypic subgroups identified using cluster analysis according quantitative CT features differed substantially in survival. Furthermore, we identified that the developed formula could provide important information about extent of fibrosis and emphysema with normal lung function in patients with IPF.

Although IPF is considered an incurable disease with poor prognosis, it has a variety of natural courses, with rapid decline, slower progression, or relative stability [19]. It is difficult to predict prognosis because of the diversity of clinical courses in IPF, and studies have sought to find simple and accurate prognostic factors [3]. Disease extent on CT has been used as a prognostic determinant of IPF. Visually stratifying the extent of honeycombing on CT may help predict prognosis in IPF with emphysema [20]. However, visual scoring of IPF by radiologists shows high interobserver variability and is somewhat subjective. Quantitative CT analysis of lung fibrosis is less time-consuming, more objective, and highly reproducible [1]. Some studies have shown that automated quantification is comparable or superior to visual scoring for evaluating pulmonary function in patients with IPF [21, 22]. A reticular opacity less than 22.05% in extent by automated quantitative assessment of regional CT pattern is related to stability in IPF [10]. Areas of increased lung attenuation via quantitative analysis on CT are associated with increased mortality, and quantitative analysis may be a clinically useful risk stratification tool in patients with interstitial lung disease [23].

The GAP (gender, age, and physiology) model predicts mortality in patients with IPF [24, 25]. Ley et al.[4] identified a CT-GAP model that replaces the DLco with a quantitative CT fibrosis score, with a comparable performance. Based on these studies, a computed-based CT image analysis was performed in patients with IPF, and some cases have already utilized the analysis in clinical practice at specialist centers [1]. In a recent study, stratifying ILD patients with a cluster analysis was predictive of disease progression and survival [15]. Therefore, we carried out a cluster analysis with quantitative features of fibrosis and emphysema on CT, classified the discrete clusters, and then assessed association with prognosis. The first cluster was composed of subjects with high FVC, high GGO, and low honeycombing and emphysema. Subjects with severe honeycombing, reticulation, and emphysema were predominantly grouped into the latter two clusters.

We found that prognosis was the best in cluster 1, with a low extent of fibrosis with emphysema and high FVC. Although they were not statistically different, cluster 2 tended to have a better prognosis than cluster 3. In cluster 2, the degree of honeycombing, reticulation, and emphysema was not significantly different from cluster 3, whereas the extent of GGO was higher. In many studies, honeycombing and reticulation are defined as a CT fibrosis score, and prognosis is worse with an increasing extent of honeycombing and reticulation [4, 10, 20]. Our study found that the extent of GGO in patients with severe fibrosis may affect prognosis. In IPF patients, GGOs should be divided into two categories including acute inflammation and chronic fibrosis. GGOs are admixed with reticulation and honeycombing, and these GGOs should be regarded as fibrotic process [6]. However, extensive pure GGOs in the non-fibrotic areas of the lung suggests acute exacerbation or infection. Our study shows active inflammation in cluster 1 and GGOs associated with reticulation in cluster 3. Furthermore, Moon et al.[26] showed that GGO ratio correlates with survival in patients with diffuse ILD. Therefore, the increased extent of GGO associated with acute inflammation or fine fibrosis has an adverse effect on patient prognosis.

PFT is commonly used as a biomarker associated with prognosis. FVC with high precision and reproducibility is linked to disease progression and mortality in IPF, and FVC decreases by an average of 150–250 mL per year in untreated IPF patients [27]. However, we found that FVC was similar between cluster 2 with severe fibrosis and cluster 1 with low fibrosis. The two clusters were significantly different in the extent of emphysema as well as fibrosis. Cluster 2 had the highest emphysema index. Approximately one-third of patients with IPF also have emphysema [14]. The presence of emphysema has been associated with mortality in patients with IPF; however, the results are inconsistent among studies [14, 28, 29]. Patients with IPF and emphysema show preservation of spirometric values and lung volumes despite extensive lung disease on CT and marked impairment of gas exchange, because emphysema leads to normalized lung volume as the compliance increased by emphysema compensates physically for the decreased compliance caused by fibrosis [11, 30]. A previous study showed that FVC is negatively correlated with extent of fibrosis, but positively correlated with emphysema [10]. According to Cottin et al.[31], FVC measurement is not appropriate for monitoring disease progression in IPF patients with emphysema extent greater than or equal to 15% on CT. Therefore, FVC does not accurately reflect disease in patients with IPF and should not be considered as an endpoint.

To overcome limitation of FVC in patients with IPF and emphysema, we developed a formula to encapsulate both fibrosis and emphysema based on the results of automated quantitative assessment of regional CT patterns: 1.5670—FS(%) * 0.04737—emphysema index*0.00304. The accuracy and TPR of the formula were 0.67 and 0.71, respectively, and this means that the spirometric results of about 71% patients with IPF and PFT normal pattern which have the score greater than 0 can be interpreted as normal due to coexistence of pulmonary fibrosis and emphysema as significant extent. However, in case of the patient with IPF which has the score greater 0 with severe fibrosis and high emphysema index, there are two cases; 1) the patient with IPF and PFT normal pattern, 2) the patient with IPF and PFT restrictive pattern. Both cases mean that the spirometric result is misinterpreted as normal. Thus based on the application of our formula, PFT results can be more cautiously interpreted while considering fibrosis and emphysema on CT in patients with IPF.

This study has several limitations. First, chest CT scans were not obtained using the same CT scanner and protocol because of the retrospective study design. Texture-based quantification can be influenced by dose, CT values, and reconstruction algorithms. We excluded 751 patients with standard kernel, low dose, or no thin-section protocols to minimize these effects. Second, in Consensus clustering, the numbers of patients in clusters 2 and 3 were small, and cluster 1 with mild fibrosis contained the majority. These small numbers were due to the exclusion of many patients with IPF in order to minimize the effect of differences in CT quantification, resulting in a study population of only 205 patients. Third, complications such as pulmonary hypertension may affect the survival of patients with IPF. Therefore, the quantitative CT features and cluster analysis we conducted may not represent mortality due to other complications. Fourth, this investigation was conducted at a single center, and so our results are not conclusive and require validation by prospective studies in a larger cohort.

## Conclusion

In conclusion, quantitative CT features related to both fibrosis and emphysema were used to classify cases into three discrete subgroups with unique radiologic phenotypes and different survival. Prognosis worsens as the extent of quantitative CT fibrosis increases, and the extent of GGO in patients with severe fibrosis may affect prognosis. In addition, cluster analysis showed that emphysema could affect FVC. The relative extent of quantitative emphysema and fibrosis on CT could help to predict lung function based on the developed formula.

## Supporting information

S1 Dataset Dataset of patients with IPF. (XLSX)
