# Correction: Predicting clinical outcome with phenotypic clusters using quantitative CT fibrosis and emphysema features in patients with idiopathic pulmonary fibrosis

**DOI:** 10.1371/journal.pone.0218223

**Published:** 2019-06-05

**Authors:** So Hyeon Bak, Hye Yun Park, Jin Hyun Nam, Ho Yun Lee, Jeong Hyun Lee, Insuk Sohn, Man Pyo Chung

There is an error in affiliation 4 for authors Hye Yun Park and Man Pyo Chung. The correct affiliation 4 is: Division of Pulmonary and Critical Care Medicine, Department of Medicine, Samsung Medical Center, Sungkyunkwan University School of Medicine, Seoul, Republic of Korea.
